# Decreased Snow Cover Stimulates Under-Ice Primary Producers but Impairs Methanotrophic Capacity

**DOI:** 10.1128/mSphere.00626-18

**Published:** 2019-01-09

**Authors:** Sarahi L. Garcia, Anna J. Szekely, Christoffer Bergvall, Martha Schattenhofer, Sari Peura

**Affiliations:** a Department of Ecology and Genetics, Limnology, Uppsala University, Uppsala, Sweden; b Department of Forest Mycology and Plant Pathology, Science for Life Laboratory, Swedish University of Agricultural Sciences, Uppsala, Sweden; Department of Energy Joint Genome Institute

**Keywords:** climate change, greenhouse gas, lakes, methane, methanotrophs, microorganisms, primary production, snow cover

## Abstract

Climate change scenarios anticipate decreased spring snow cover in boreal and subarctic regions. Forest lakes are abundant in these regions and substantial contributors of methane emissions. To investigate the effect of reduced snow cover, we experimentally removed snow from an anoxic frozen lake. We observed that the removal of snow increased light penetration through the ice, increasing water temperature and modifying microbial composition in the different depths. Chlorophyll *a* and *b* concentrations increased in the upper water column, suggesting activation of algal primary producers. At the same time, *Chlorobiaceae*, one of the key photosynthetic bacterial families in anoxic lakes, shifted to lower depths. Moreover, a decrease in the relative abundance of methanotrophs within the bacterial family *Methylococcaceae* was detected, concurrent with an increase in methane concentration in the water column. These results indicate that decreased snow cover impacts both primary production and methane production and/or consumption, which may ultimately lead to increased methane emissions after spring ice off.

**IMPORTANCE** Small lakes are an important source of greenhouse gases in the boreal zone. These lakes are severely impacted by the winter season, when ice and snow cover obstruct gas exchange between the lake and the atmosphere and diminish light availability in the water column. Currently, climate change is resulting in reduced spring snow cover. A short-term removal of the snow from the ice stimulated algal primary producers and subsequently heterotrophic bacteria. Concurrently, the relative abundance of methanotrophic bacteria decreased and methane concentrations increased. Our results increase the general knowledge of microbial life under ice and, specifically, the understanding of the potential impact of climate change on boreal lakes.

## Abstract (precis)

Small lakes are an important source of greenhouse gases in the boreal zone. These lakes are severely impacted by the winter season, when ice and snow cover obstruct gas exchange between the lake and the atmosphere and diminish light availability in the water column. Currently, climate change is resulting in reduced spring snow cover. A short-term removal of the snow from the ice stimulated algal primary producers and subsequently heterotrophic bacteria. Concurrently, the relative abundance of methanotrophic bacteria decreased and methane concentrations increased. Our results increase the general knowledge of microbial life under ice and, specifically, the understanding of the potential impact of climate change on boreal lakes.

## INTRODUCTION

Small forest lakes are a typical feature of boreal and subarctic regions (1). These small water bodies with high organic loads are hot spots in the carbon cycle and one of the most prominent environmental sources of greenhouse gas emissions in these regions (2, 3). The microorganisms inhabiting such lakes are the main drivers of these biogeochemical processes (4). Microbial community structure and functioning are strongly impacted by environmental conditions and seasonality. Winter conditions have a particularly strong impact on life in the lake, as ice and snow cover isolate the lake water column from the surrounding environment (5).

One of the most striking impacts of ice and snow cover is the impairment of light availability in the lake. Food webs in most lakes are based on primary production by algae, which provide substrates and oxygen for lake bacteria and serve as important food sources for zooplankton. In wintertime, decreased light availability curtails photosynthesis beneath the ice (6–8), while ice cover also inhibits oxygen transfer from the atmosphere, both contributing to lower under-ice oxygen concentrations. Moreover, aerobic organisms consume residual oxygen in the water beneath the ice (9), leading to a hypoxic-to-anoxic gradient from the lake surface to bottom waters. Anoxic conditions facilitate anaerobic processes, such as methanogenesis, and decrease methanotrophic capacity (10). As a result, methane accumulates under ice and is emitted during ice-break in the spring (11, 12). At the same time, some organisms might benefit from winter conditions, as previously shown for taxa within the bacterial phylum *Verrucomicrobia* (13). To date, however, studies that address winter conditions are lagging behind open water season research (14).

Currently, climate change is resulting in altered seasonal patterns in subarctic regions (15) and patterns of snow cover worldwide (16). In the Northern Hemisphere, these changes include sudden extreme conditions, as seen in northern Scandinavia during the 2007/08 winter, when snow cover loss occurred abruptly due to a warming event (17). Long-term decreases in spring snow cover have also been observed (16). As snow cover is a major determinant of the amount of light under ice in lakes (5, 18), changes in its thickness and distribution may directly or indirectly affect microbial communities and their activities. For example, cell division among diatoms adapted to low-light conditions—such as those under snow-covered ice—is inhibited when exposed to higher light levels (18). In addition, algal blooms under ice have been shown to be controlled by snow cover (19). Since algae are the sole source of oxygen under ice, a fluctuation in snow cover likely has an impact on algal processes, on other organisms that interact directly and indirectly with algae, and on interactions between nonalgal organisms. We conducted a snow removal experiment on a frozen, oxygen-stratified lake to address the impact of snow cover variation on conditions in the water column and on microbial organisms, with emphasis on methane cyclers and primary producers.

We expected that removal of snow cover would deepen light penetration (5, 18) and hypothesized that this would stimulate primary production. Further, we hypothesized that higher photosynthetic activity would improve oxygen availability and impact the aerobic bacteria in general and specifically increase methanotrophic activity.

## RESULTS

**FIG 1:**
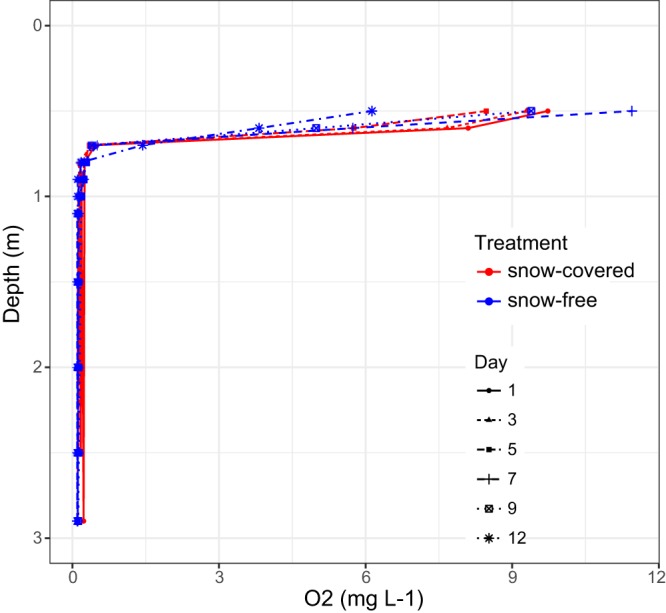
Oxygen concentration (in milligrams per liter) under the ice during the experiment.

Our sampling scheme included six time points and six depths (0.65, 1.00, 1.35, 1.85, 2.35, and 0.5 m above the sediment surface at each location—either 2.55 or 2.85 m) measured from the top surface of the ice layer). We sampled a vertical profile of the lake on three occasions before the snow removal and three times after snow removal, with 1 day between each sampling, except for the last sampling that was 2 days after the previous sampling. Thus, the total duration of the experiment was 2 weeks. The snow depth on the frozen lake was 18 to 21 cm through the experiment, and ice thickness was approximately 50 cm, consisting of mainly black ice with a few centimeters of white ice at the top. Prior to the snow removal, the lake had a shallow oxic layer (epilimnion), with a steep oxygen depletion layer from 0.6 to 0.7 m, where the oxygen concentration was under 0.5 mg liter^−1^ (Fig. 1).

**FIG 2:**
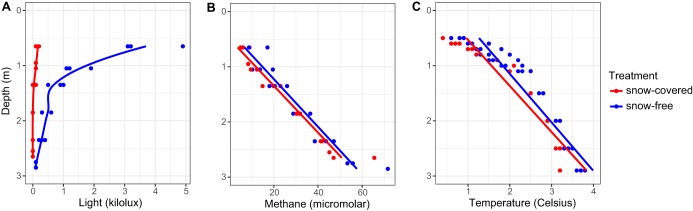
Light intensity (A), methane concentration (B), and temperature (C) in the lake water column during the experiment.

**FIG 3:**
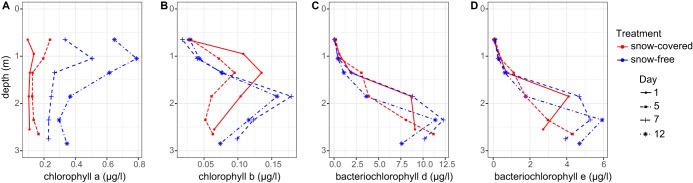
Concentrations of chlorophyll *a* (A), chlorophyll *b* (B), bacteriochlorophyll *d* (C), and bacteriochlorophyll *e* (D) in the lake water column during the experiment. The concentrations are shown in micrograms per liter.

We observed a significant light intensity increase at all depths of the lake after the snow removal, although the extent of the change decreased with increasing depth (Fig. 2 and Table 1). The water temperature also increased throughout the water column after the snow was removed. Moreover, chlorophyll *a* concentration increased in the three upper layers (0.65 to 1.35 m), whereas chlorophyll *b* increased in the three bottom layers (1.85 to 2.85 m). Furthermore, the concentration of bacteriochlorophyll *d* and *e* appeared to increase at a depth of 2.35 m (Fig. 3). On the basis of these results, we presume that light had a direct effect on the phytoplankton, increasing their chlorophyll production, and likely affecting their photosynthetic activity and oxygen production. After the snow removal, an increase in oxygen concentration in the layer directly under ice was observed, which rapidly decreased over time (Fig. 1). However, differences in oxygen concentration were not statistically significant (Table 1).

**TABLE 1:** Impact of treatment and depth on environmental and chemical factors and on the bacterial community^*a*^

Factor or bacterial family^*b*^	Between depths		Within depths	
	Treatment	Depth	Treatment	Treatment × depth
Light	**0.012**	**<0.001**	**0.025**	0.169
Temperature	0.555	**0.005**	**0.003**	0.547
Oxygen	0.309	**0.012**	0.346	**0.014**
Methane	**0.012**	**<0.001**	**0.023**	0.169
*Methylococcaceae*				
DNA	0.292	**0.033**	**0.009**	0.121
RNA	*0.094*	**0.012**	0.277	0.259
*Comamonadaceae*				
DNA	0.364	*0.067*	**0.005**	0.114
RNA	0.113	**0.023**	**<0.001**	0.970
*Flavobacteriaceae*				
DNA	**0.040**	**0.004**	0.653	0.995
RNA	0.240	*0.073*	0.615	*0.093*
*Chlorobiaceae*				
DNA	0.181	**0.034**	0.680	0.321
RNA	0.084	**0.014**	0.635	**0.021**

a The results of repeated measures two-way ANOVA for testing the impact of treatment and depth to light intensity, temperature, concentration of oxygen and methane, and the relative abundance of dominant taxonomic bacterial groups in the lake water column. Significant results (*P* < 0.05) are shown in bold type, while marginal significant results (*P* < 0.1) are shown in italic type.

b The dominant bacterial group or family and whether the bacterial community analysis was based on DNA or RNA are shown.

As expected, prior to snow removal, methane concentrations increased with depth. However, in contrast to our hypothesis, methane concentrations were significantly higher after the snow removal (Fig. 2 and Table 1). There was a depth-related decrease in phosphate concentrations at 0.65, 1.35, 1.85, and 2.35 m after the treatment, but none of the nutrient concentrations changed significantly during the experiment (see Table S1 in the supplemental material).

10.1128/mSphere.00626-18.2 TABLE S1 Concentrations (and standard deviations) of nitrate, nitrite, ammonium, phosphate, and sulfate in Lake Lomtjärn during the experiment. Download Table S1, DOCX file, 0.1 MB. Copyright © 2019 Garcia et al. 2019 Garcia et al. This content is distributed under the terms of the Creative Commons Attribution 4.0 International license.

Overall, the 11 most abundant operational taxonomic units (OTUs) each contained at least 1% of the sequences. Together, these 11 OTUs accounted for 32% of the sequences. The most abundant OTU belonged to the *Chlorobiaceae* family, while the second and third most abundant OTUs belonged to the *Methylococcaceae* family. Snow removal had a significant effect on the total community composition at the DNA level but not at the RNA level (permutational analysis of variance [PERMANOVA], p_DNA_ = 0. 0.008, p_RNA_ = 0. 073). Depth was an important driver of community composition (PERMANOVA, p_DNA_ < 0.001, p_RNA_ < 0.001). The major microbial groups in water at a depth of 0.65 m were *Betaproteobacteria* (26% before and 32% after snow removal in relative abundance of DNA-based bacterial community composition; 30% before and 46% after snow removal in RNA-based community) and *Gammaproteobacteria* (23% before and 7% after snow removal in DNA; 37% before and 18% after snow removal in RNA) (see Fig. S1 in the supplemental material). Most of the gammaproteobacterial organisms observed in the water column belonged to the family *Methylococcaceae*, which consists solely of methane-consuming bacteria.

10.1128/mSphere.00626-18.1 FIG S1 Composition of the bacterial community according to the proportions of 16S rRNA genes in the DNA (top panel) and RNA (bottom panel) from the surface of the lake (depth 1) to the bottom (depth 6). Download FIG S1, PDF file, 0.01 MB. Copyright © 2019 Garcia et al. 2019 Garcia et al. This content is distributed under the terms of the Creative Commons Attribution 4.0 International license.

**FIG 4:**
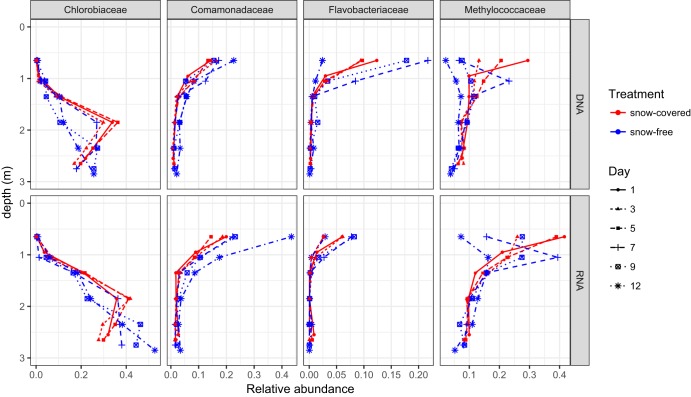
Relative abundance of phototrophic *Chlorobiaceae*, heterotrophic bacteria (*Comamonadaceae* and *Flavobacteriaceae*), and methanotrophic *Methylococcaceae* in the lake water column during the experiment.

Changes in both DNA and RNA were observed for most of the groups that exhibited the most substantial alterations posttreatment. Among the most abundant taxonomic groups, we observed an increase in the relative abundance of the *Comamonadaceae* and *Flavobacteriaceae* families after the snow removal, which were dominant groups in the uppermost layer. Concurrently, the abundance of *Methylococcaceae* decreased following the experimental treatment (Fig. 4 and Table 1). Finally, the maximum abundance of *Chlorobi*, a dominant microbial group in the anoxic compartment, appeared to have shifted to deeper layers (Fig. 4).

## DISCUSSION

Our results show that the impact of decreased snow cover on the lake microbiome and metabolism is a complex interaction between biotic and abiotic lake characteristics. Increases in chlorophyll concentrations throughout the water column confirmed our first hypothesis, which postulated that the increase of light following the snow removal would stimulate the primary producers. However, our second hypothesis was verified only partially, as the increase in oxygen concentration that we observed did not persist. Nevertheless, we detected changes in the relative abundances of heterotrophic and methanotrophic bacteria following snow cover removal. Finally, we reject our third hypothesis, which is the anticipated increase of methanotrophic activity with decreased snow cover.

**FIG 5:**
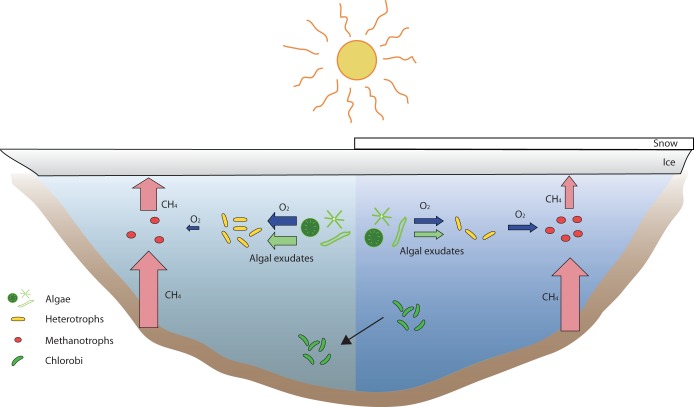
A conceptual figure visualizing the dynamics of algal primary producers, heterotrophic bacteria, methanotrophic bacteria, and bacterial phototrophs (*Chlorobi*). The right side illustrates the conditions found in the lake with ice and snow cover. The left side illustrates how the conditions changed after the removal of snow, such as increase in light and increased methane throughout the water column. In the first depth sampled, methanotrophs decreased after snow removal, while heterotrophs increased. Moreover, the *Chlorobi* populations shifted to lower depths in the water column.

Here, the results show that the algal community was stimulated, producing substrates that likely enhance the activities of aerobic, heterotrophic bacteria within the families *Comamonadaceae* and *Flavobacteriaceae* (Fig. 5). Members of these families are aerobic heterotrophs capable of thriving on increased availability of oxygen and organic compounds originating from increased activity of the primary producers (20–22). The immediate oxygen drawdown may be due to decomposition of algal exudates and other alga-derived compounds by heterotrophic bacteria (Fig. 5). In accordance with this assumption, bacterial abundance almost doubled in the upper layer of the lake, concurrent with the oxygen utilization (see Table S2 in the supplemental material). At the same time, the relative abundance of methanotrophic bacteria decreased (Fig. 5). Many methanotrophs are known to have low growth rates (23); hence, heterotrophs potentially outgrew the methanotrophs due to increased availability of algal substrates and oxygen. Other explanations for the decrease of methanotrophs could be low phosphate concentration, which has been linked to impaired methanotrophic activity (24). Increased algal activity in the lake after snow removal may have resulted in phosphate depletion, impeding the growth of methanotrophs. Alternatively, increased primary production could have contributed more substrate to methanogenesis in surface sediments, likely increasing the concentration of methane in the water column through diffusion (12). Decreased methanotrophy and increased methanogenesis are consistent with the increased methane concentrations after the snow removal. However, the specific reasons for the reduced relative abundances of methanotrophs, as well as the impact of a longer period of snow-free ice cover on methanotrophic communities, require further investigation.

10.1128/mSphere.00626-18.3 TABLE S2 Bacterial abundance during the experiment (number of bacteria per milliliter). Download Table S2, DOCX file, 0.04 MB. Copyright © 2019 Garcia et al. 2019 Garcia et al. This content is distributed under the terms of the Creative Commons Attribution 4.0 International license.

Several experimental features should be taken into consideration in interpreting our results. One important factor to consider is the short duration of our experiment, which makes our results mainly applicable to short-term effects of snow depletion. However, as under-ice algal growth rates can be comparable to those of blooming cyanobacteria (25, 26), and bacterial communities beneath ice are capable of swiftly responding to environmental change (27), we were able to detect shifts in communities related to decreased snow cover within the narrow experimental window. The extent and nature of community shifts in response to changed conditions on a longer time frame must be investigated through other experimental approaches. Another important factor to consider is that we estimated the photosynthetic activity using chlorophyll concentrations, which is an indirect way to measure primary production. However, chlorophyll measurements are widely used as an estimator of photosynthetic activity. In the ocean, for example, chlorophyll concentrations reliably reflect the photosynthetic potential of primary producers (28, 29). Moreover, the presence of bacterial photosynthetic community members was detected not only by bacteriochlorophyll but also by DNA and RNA amplicon sequencing (i.e., *Chlorobi*; Fig. 3 and 4), suggesting active photosynthesis in the ice-covered lake.

*Chlorobi* is a common community member in boreal lakes (30–32), such as our study lake, and may contribute substantially to inorganic carbon assimilation in this environment (30). Because of the abundance of bacteriochlorophylls in their antenna complexes, some of these green sulfur bacteria are able to grow at extremely low light levels (1 to 10 nmol photons m^−2^ s^−1^), under which no other types of chlorophototrophs can grow (33, 34). Decrease in the abundance of *Chlorobi* after snow removal at a depth of 1.85 m was likely due to light intensity increase, making conditions more favorable to organisms that use chlorophyll *a* and *b* (Fig. 1). Moreover, an increase of *Chlorobi*—in DNA- and RNA-based relative abundances—was observed in deeper layers of the lake (Fig. 4). This finding, coupled with the increase in bacteriochlorophyll *d* and *e* around 2.35 m, suggests that decreased snow cover alters the taxonomical composition of the primary producers of the lake, pushing the optimal conditions for *Chlorobi* to lower depths of the lake. Changes in primary producers may have implications for lake carbon balance, owing to likely shifts in the efficiency of carbon dioxide uptake and microbial interactions; however, further studies are needed to verify these hypothesized mechanisms.

For the bacterial community in general, depth was an important driver of community composition, likely explained by decreasing oxygen concentrations down the water column. A decrease in oxygen typically cooccurs with a shift in redox potential, both of which are key factors structuring bacterial communities (35, 36). The dominant community members in the lake were the same as previously reported for similar lakes (30–32, 37), suggesting that our results likely apply to a large portion of boreal lakes.

### Conclusions.

Our results suggest that decreased snow cover can be expected to impact total lake metabolism and potentially increase methane emission after ice-break. Climate change may additionally lead to shortened ice cover period with earlier ice-off (14), the implications of which have yet to be investigated in boreal lakes. A shorter ice cover period would likely coincide with changes in snow dynamics, and the end results will highly depend on the response of microbial communities. Our results are representative of short-term impacts of decreased snow; the consequences of this phenomenon across other boreal lakes and over longer time scales must be investigated in future studies. In any case, our observations strongly suggest that decreased snow in ice-covered lakes has the immediate potential to increase methane concentrations in the water column, which could lead to increased methane emissions to the atmosphere once the ice melts.

## MATERIALS AND METHODS

The experiment was conducted in March 2016 on Lake Lomtjärnan (63°20'56.9"N 14°27'28.3"E), a small forest lake located in Krokom, Sweden. The surface area of the lake is about 1 ha, and the maximum depth is 3.5 m. The lake is located on a mire surrounded by a coniferous forest. The lake has characteristics shared by millions of lakes in the boreal region, such as decreasing oxygen concentration and light intensity from the surface to the bottom and a nutrient and temperature gradient (see Table S1 in the supplemental material). The lake is covered with ice during the winter months, approximately from November to April. The experiment consisted of two parts: first, the lake was monitored while there was still snow cover on the ice surface, and second, the impact of snow cover removal—from an area of approximately 400 m^2^ above the deepest part of the lake—was observed. Snow was removed manually using snow shovels on day 6 of the monitoring. Under-ice water was sampled by drilling holes through the ice at various locations to ensure that the sampled parameters and microbial communities were as close to an unaltered state as possible, beyond the changes potentially imposed by the experimental snow removal. After sampling, each hole was filled and covered with snow to limit oxygen and light penetration to the water column.

Light intensity and temperature were measured with 18 HOBO loggers (HOBO Pendant temperature/light 64K data logger, Onset Computer Corporation, USA). On the first day of the experiment, loggers were placed under the ice, measuring parameters from the bottom of the ice to the bottom of the lake every 0.1 to 0.75 m; the loggers were maintained at the same place throughout the duration of the experiment. Laterally, the sensors were approximately 50 cm from the hole in the ice. The light values are presented as daily averages for the time between sunrise and sunset (approximately 10 AM to 3 PM).

During each sampling occasion, samples were taken to measure chemical parameters (chlorophyll *a* and *b*, nitrite, nitrate, phosphate, sulfate, ammonia, fluoride, chloride, oxygen, methane, and carbon dioxide) and DNA- and RNA-based community analyses. Oxygen concentration was measured with YSI 55 combined temperature and oxygen probe (Yellow Springs Instruments, Yellow Springs, OH, USA). Nutrients were measured by standard methods.

For DNA and RNA, samples were taken with Sterivex filters (Millipore, Billerica, MA, USA). Filtration for RNA was limited to 15 min, whereas filtration for DNA continued until the filter was clogged. Samples were taken from five different depths on day 1, as well as from six different depths on days 3 and 5 (before the snow removal), and days 7, 9, and 12 (after the snow removal).

Chlorophyll pigments were extracted on ice, in tubes containing 2 ml of 90% acetone, using an ultrasonic bath; these extractions occurred overnight at −20°C. Samples were then centrifuged (3,000 × 10 min at 4°C) and filtered (0.45-μm syringe filters). Extracts were analyzed using HPLC on an Agilent 1100 Series HPLC system (Agilent Technologies, Waldbronn, Germany) fitted with three RP-18e Chromolith Performance columns (100 by 4.6 mm) (Merck, Darmstadt, Germany) connected in series. The flow rate was 1.4 ml/min, and the gradient program used is described in detail elsewhere (38). The column temperature was 25°C, and the injection volume was 100 μl (70 μl sample plus 30 μl 0.5 M ammonium acetate). Absorbance was measured with a diode array detector between 300 to 800 nm (resolution 2 nm and slit with 4 nm). Chlorophyll *a* and *b* were identified and quantified using standard solutions (DHI Laboratory Products, Hoersholm, Denmark); bacteriochlorophylls were separately identified using previously published chromatograms, spectra, and extinction coefficients (39–43). Methane concentration was analyzed as described previously (44), except that room air was used instead of nitrogen for the headspace. The methane concentration was also analyzed from room air and subtracted from the final gas concentrations.

DNA and RNA were coextracted using a phenol-chloroform method (45) with modifications (30). RNA was then transcribed into cDNA as previously described (46) using RevertAid H Minus First Strand cDNA synthesis kit (Thermo Scientific). Thereafter, RNA and DNA samples were amplified for bacterial 16S rRNA genes using primers 341r and 805f (47). The PCR conditions were set as previously described (48). The samples were then pooled in equimolar amounts and sequenced with Illumina MiSeq at Science for Life Laboratory (Uppsala, Sweden). The resulting 2.5 million sequences were processed using mothur (49) as described elsewhere (50), except that operational taxonomic unit (OTU) clustering was done using abundance-based greedy clustering.

The effects of snow removal and water depth were tested with two-way repeated-measures analyses of variance (RM-ANOVA) for the environmental parameters and by two-way nested permutational ANOVA (PERMANOVA) with 9,999 permutations for the community composition data. Univariate data were log transformed to fulfill the assumptions of RM-ANOVA. All statistical analyses were done in R version 3.4.3 (51). Packages phyloseq, vegan, and ggplot2 were used (52–54).

### Accession number(s).

Raw sequences have been submitted to the European Nucleotide Archive (ENA) under accession numbers ERS2597919 to ERS2597988.
