# Supplementary material for: Decreased Snow Cover Stimulates Under-Ice Primary Producers but Impairs Methanotrophic Capacity
Source: mSphere. 2019 Jan 9;4(1):e00626-18. doi: 10.1128/mSphere.00626-18 (PMC6327105; doi:10.1128/mSphere.00626-18)
Supplement: TABLE S1 [file mSphere.00626-18-st001.docx]

Table S1. The concentration (and standard deviation) of nitrate, nitrite, ammonium, phosphate and sulphate in Lake Lomtjärn during the experiment.

| Depth (m) | NO_3_ (mg L^-1^) | NO_2_ (mg L^-1^) | NH_4_ (mg L^-1^) | PO_4_ (mg L^-1^) | SO_4_ (mg L^-1^) |
| --- | --- | --- | --- | --- | --- |
| 0.65 | 343.0 (170.6) | 9.2 (12.2) | 187.0 (81.6) | 1.5 (1.6) | 3.5 (0.7) |
| 1.00 | 162.1 (119.1) | 6.0 (6.8) | 332.6 (75.5) | 1.0 (0.9) | 4.0 (0.6) |
| 1.35 | 44.5 (39.8) | 0.7 (1.7) | 353.8 (98.2) | 0.3 (0.4) | 3.8 (0.1) |
| 1.85 | 25.1 (8.4) | 0 | 548.7 (24.7) | 0.3 (0.6) | 3.3 (0.2) |
| 2.35 | 26.4 (9.6) | 0 | 556.2 (135.8) | 1.1 (1.7) | 2.5 (0.2) |
| 0.50 m above the sediment | 16.1 (4.8) | 0 | 631.6 (145.2) | 1.5 (1.8) | 2.2 (0.2) |
