# Supplementary figures and images for: Decreased Snow Cover Stimulates Under-Ice Primary Producers but Impairs Methanotrophic Capacity
Source: mSphere. 2019 Jan 9;4(1):e00626-18. doi: 10.1128/mSphere.00626-18 (PMC6327105; doi:10.1128/mSphere.00626-18)

# Class Composition of Lake Lomtjaernen Bacterial Communities Grouped by Depth

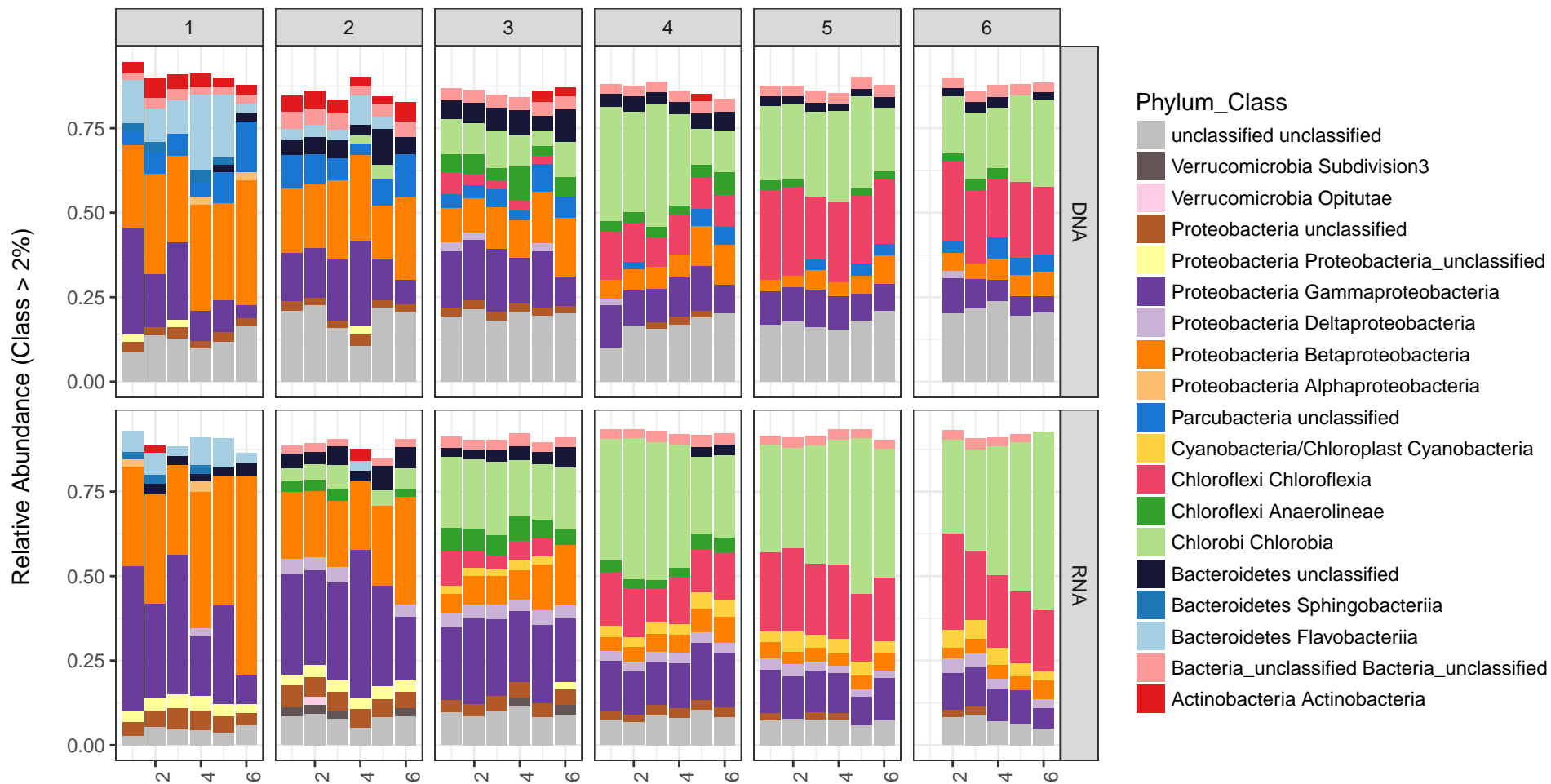

Supplement: FIG S1 [file mSphere.00626-18-sf001.pdf]
