# Supplementary material for: Decreased Snow Cover Stimulates Under-Ice Primary Producers but Impairs Methanotrophic Capacity
Source: mSphere. 2019 Jan 9;4(1):e00626-18. doi: 10.1128/mSphere.00626-18 (PMC6327105; doi:10.1128/mSphere.00626-18)
Supplement: TABLE S2 [file mSphere.00626-18-st002.docx]

Table S2. The bacterial abundance during the experiment (ml^-1^)

| Depth | Day 1 | Day 2 | Day 3 | Day 4 | Day 5 | Day 6 |
| --- | --- | --- | --- | --- | --- | --- |
| 1 | 1.23E+05 | 1.74E+05 | 1.94E+05 | 2.33E+05 | 2.97E+05 | 3.25E+05 |
| 2 | 1.95E+05 | 2.62E+05 | 4.96E+05 | 1.83E+05 | 3.47E+05 | 2.20E+05 |
| 3 | NA | 2.66E+05 | 3.30E+05 | 4.19E+05 | 2.50E+05 | 2.08E+05 |
| 4 | 3.00E+05 | 3.25E+05 | 3.83E+05 | 2.81E+05 | 2.31E+05 | 3.05E+05 |
| 5 | NA | 3.25E+05 | 3.53E+05 | 1.91E+05 | 2.29E+05 | 2.59E+05 |
| 6 | 3.52E+05 | 2.24E+05 | 3.32E+05 | 2.61E+05 | 2.33E+05 | 2.70E+05 |
